# Pain in Neurodegenerative Disease: Current Knowledge and Future Perspectives

**DOI:** 10.1155/2016/7576292

**Published:** 2016-06-05

**Authors:** Marina de Tommaso, Lars Arendt-Nielsen, Ruth Defrin, Miriam Kunz, Gisele Pickering, Massimiliano Valeriani

**Affiliations:** ^1^Neurophysiopathology of Pain Section, SMBNOS Department, Bari Aldo Moro University, Bari, Italy; ^2^Center for Sensory-Motor Interaction, Aalborg University, Aalborg, Denmark; ^3^Department of Physical Therapy, Sackler Faculty of Medicine, Tel-Aviv University, Tel-Aviv, Israel; ^4^Department of General Practice, Section Gerontology, University Medical Center Groningen, Groningen, Netherlands; ^5^CHU Clermont-Ferrand, Centre de Pharmacologie Clinique, Clermont-Ferrand, France; ^6^Inserm, CIC 1405, Neurodol 1107, 63003 Clermont-Ferrand, France; ^7^Division of Neurology, Ospedale Pediatrico Bambino Gesù, IRCCS, Rome, Italy

## Abstract

Neurodegenerative diseases are going to increase as the life expectancy is getting longer. The management of neurodegenerative diseases such as Alzheimer's disease (AD) and other dementias, Parkinson's disease (PD) and PD related disorders, motor neuron diseases (MND), Huntington's disease (HD), spinocerebellar ataxia (SCA), and spinal muscular atrophy (SMA), is mainly addressed to motor and cognitive impairment, with special care to vital functions as breathing and feeding. Many of these patients complain of painful symptoms though their origin is variable, and their presence is frequently not considered in the treatment guidelines, leaving their management to the decision of the clinicians alone. However, studies focusing on pain frequency in such disorders suggest a high prevalence of pain in selected populations from 38 to 75% in AD, 40% to 86% in PD, and 19 to 85% in MND. The methods of pain assessment vary between studies so the type of pain has been rarely reported. However, a prevalent nonneuropathic origin of pain emerged for MND and PD. In AD, no data on pain features are available. No controlled therapeutic trials and guidelines are currently available. Given the relevance of pain in neurodegenerative disorders, the comprehensive understanding of mechanisms and predisposing factors, the application and validation of specific scales, and new specific therapeutic trials are needed.

## 1. Introduction

Neurodegenerative diseases are going to increase in parallel to the lengthening of survival. The most common of them become more prevalent with age being accompanied by progressive motor and cognitive impairment. The management of neurodegenerative diseases as Alzheimer's disease (AD) and other dementias, Parkinson's disease (PD) and PD related disorders, motor neuron diseases (MND), Huntington's disease (HD), spinocerebellar ataxia (SCA), and spinal muscular atrophy (SMA), is mainly addressed to motor and cognitive impairment with special care to the vital functions as breathing and feeding. Many of these patients complain of painful symptoms though their origin is variable, and their presence is frequently not considered in the treatment guidelines, leaving their management to the decision of the clinicians alone. In some neurodegenerative diseases as Parkinson's disease, pain has recently been recognized as a frequent and invalidating symptom [1]. In general, pain treatment should mainly be based on its pathophysiological mechanisms. According to the International Association for the Study of Pain (IASP), pain is an unpleasant sensory and emotional experience associated with actual or potential tissue damage or described in terms of such damage [2]. Most pain syndromes are neuropathic or nociceptive in their origin. While central and peripheral neuropathic pain are caused by a lesion or disease of the central or peripheral somatosensory nervous system, respectively, the nociceptive pain arises from actual or threatened damage to nonneural tissue and is due to the activation of nociceptors [3]. Thus, nociceptive pain occurs in patients with a normally functioning somatosensory nervous system [3]. Neurodegeneration may specifically involve the somatosensory system, thus making a neuropathic origin of pain very likely, or it may affect cortical and subcortical structures involved in pain modulation. Motor impairment with muscular tone abnormalities and reduced active mobility may cause osteoarticular problems with local inflammation and nociceptive pain. In many neurodegenerative conditions, the origin of pain is complex, often multifactorial and hardly classifiable as merely neuropathic or nociceptive. In addition, there are few evidences on frequency and characteristics of pain symptoms in neurodegenerative disorders and on their impact on the disease outcome. An IASP task force [4] has revised the clinical and instrumental assessment of chronic pain as well as its therapeutic management so a systematic application of these guidelines to chronic pain in neurodegenerative diseases should be within reach. However, pain assessment may be hampered by the impairment of cognitive and motor performances so special recommendation should be provided upon approaching this important aspect of neurodegenerative diseases.

The present review focuses on chronic pain in main neurodegenerative diseases addressing the current knowledge about pain frequency and clinical features, clinical and instrumental assessment, possible pathophysiological mechanisms, and the current evidence on pain therapeutic management. Also the main limitations of the present studies and the future research direction and perspectives are considered.

We also dedicated a section to rare neurodegenerative conditions where pain was not extensively assessed.

This was a narrative review based on PubMed search by the following key words: pain, pain frequency, pain features, pain treatment and Alzheimer disease, Parkinson's disease, extrapyramidal disorders, motor neuron disease, and spino-cerebellar ataxia. There was no time limit for the research, starting form the first date reported by PubMed.

## 2. Alzheimer Disease and Other Dementias

Dementia refers to a broad category of brain neurodegenerative diseases that are accompanied by loss of ability in memory function, attention, executive function, orientation, language, and other cognitive domains as well as by changes in mood and behavior, which increase across the course of dementia. There are an estimated 35 million people with dementia in the world. The most common type of dementia is Alzheimer's disease. Other forms of dementia are vascular dementia, frontotemporal dementia, and Lewy body dementia. Given that the prevalence rate of dementia is tightly linked to ageing, the increase of ageing population is the main reason for the predicted substantial growth in the number of people affected by dementia. Not only do prevalence rates of dementia increase with age, but also the prevalence rates of pain are strongly linked to ageing [5]. Given that both the prevalence rates of dementia and pain increase with age, it appears that pain is highly common among people suffering from dementia. The difficulty is, however, that patients with dementia (particularly those in the advanced stages of the disease) are often unable to use self-report to communicate their suffering, and thus their pain is often overlooked and remains untreated [6].

### 2.1. Pain Frequency and Clinical Features

Due to their advanced age, individuals with dementia often suffer from multiple morbidities associated with pain. However, the exact pain prevalence in dementia is unknown due to the lack of self-report in this patient group as mentioned previously. Studies using observational tools to assess pain indicate that about 50% of patients with dementia living in nursing homes are suffering from pain [13, 28]. This is in line with prevalence rates reported about nursing home patients, independently of their cognitive status. Yet, the prevalence of pain among patients with dementia is associated with the severity of the condition. In fact, between 45% and 83% of the patients living in nursing homes experience acute or chronic pain [29]. Most of these patients (about 94%) were reported to suffer from persistent pain (3–6 months or more). The causes of pain among patients with dementia living in nursing homes include but are not restricted to genitourinary infections, pathologies in the musculoskeletal system [30], pressure ulcers [31], and skin diseases, with the latter of which found in 95% of the patients and described as one of the most prevalent health problems in this population [32].

Several studies have estimated the prevalence rates of pain among patients with dementia who are living at home. These prevalence rates are listed in Table 1 [7–13]. What appears evident is that the rates vary across studies, depending on whether they are based on the self-report or on the caregivers' report. Overall, at least 50% of the community dwelling patients with dementia seem to be suffering from pain. Considering the aforementioned studies, pain is highly prevalent among patients with dementia whether living in nursing homes or living at home, and thus its management required careful assessment and monitoring.

### 2.2. Clinical and Instrumental Assessment

Given the high prevalence of pain in the elderly, proper assessment of pain by observers such as health care professionals or family members is required for successful pain treatment. Due to the subjective and complex nature of the pain experience, assessing patients' pain is often a challenge. The more severe the cognitive impairment is and hence the more severe the loss of self-report is, the greater the challenge is [33]. In such instances, caregivers should rely less on self-report and more on behavioral indicators of pain. Facial expressions in particular seem to provide a valid indication of the patients' pain. Patients with dementia display the same types of facial movements in response to pain as cognitively intact individuals [34, 35]. Thus, the pain-peculiarity of their facial expression is not reduced. This finding implies that facial expressions of pain have the potential to serve as an alternative pain indicator in patients with dementia. Other means to indirectly assess pain are various behavior rating scales (for comprehensive reviews, see, e.g., Herr et al. [36] and Zwakhalen et al. [13]). However, the validation process of these scales has just started. Although the first results are promising, future studies are needed to define which items are able to discriminate between pain behavior and behaviors related to other aspects of unmet needs in patients with dementia. Within a European initiative (COST TD1005), researchers across Europe have started to investigate which behavioral items can indeed validly indicate the presence of pain in patients with different types of cognitive impairment. The process is still ongoing [37] but will hopefully be completed within the next 1-2 years.

### 2.3. Possible Pathophysiological Mechanisms

Only a few experimental studies have tried to investigate how dementia affects the processing of nociceptive information with most studies focusing on patients with Alzheimer's disease (for a comprehensive review, see Defrin et al. [38]). Alarmingly, the majority of the experimental findings seem to suggest that the processing and the experience of pain are not diminished in patients with mild-to-moderate forms of dementia. On the contrary, the pain experience might even be enhanced. It has been reported that patients with dementia respond to noxious stimulation with more enhanced facial responses [34, 35] and pain withdrawal reflexes [35] compared with cognitively intact peers. Functional magnetic resonance imaging (fMRI) studies showed that brain activity in response to noxious stimulation is preserved and even elevated in patients with mild forms of Alzheimer's disease [39, 40] corroborating findings based on facial and reflexive expressions. Using evoked related potentials (ERPs), one study found no difference in peak amplitude between patients and controls [41] and one study failed to induce pain-evoked potentials in the subgroup of patients with severe dementia [42]. The widespread brain damage occurring in the course of dementia might possibly affect descending pain modulation pathways most strongly in mild-to-moderate stages of dementia, which in turn lead to reduced inhibitory control over the pain system and increased pain processing. In later stages of dementia, the ascending pain pathways might be affected more severely, resulting in reduced pain processing. However, this is only speculative since research on patients at severe stages of dementia is lacking.

### 2.4. Current Evidence on Pain Therapeutic Management

Recent reviews on pain management in patients with dementia point to a severe lack of effective assessment and treatment in different clinical settings and to the conclusion that even today patients with dementia are still undertreated for pain compared with nondemented elderly individuals [43, 44]. However, this seems to be slowly changing. Two studies from Scandinavia [45, 46] found that patients with dementia received even more analgesic drugs (mostly paracetamol) compared with those without dementia. The increased dosage was administered even though the patients reported pain less frequently, and the prevalence of the pain related diagnoses was similar compared with persons without dementia. Beyond being very promising, these results clearly suggest that the research findings of the last decades—which reported an undertreatment of pain in dementia—have already impacted the clinical practice and have led to an intensification of pain management in this frail patient group.

### 2.5. Main Limitations of the Present Studies and Future Directions

In the last two decades, much effort has been invested to better understand how dementia affects the pain processing as well as its assessment. Up to now, an impressive number of pain behavior rating scales have been developed trying to assess pain based on nonverbal behavior. However, the validity, usability, and feasibility of these scales are still unsatisfactory, and they are not often used in the clinical practice. Moreover, dementia is a very broad concept that includes not only various types of neurodegenerative processes affecting different brain areas but also different degrees of cognitive decline. Most studies do not differentiate between different types or stages of dementia. Moreover, research investigating patients with dementia at the last stage of the disease is still mostly lacking, and, therefore, we do not know how pain processing might be altered in these very fragile patients. Moreover, it is an alarming fact that patients with dementia are still excluded from high quality randomized controlled trials of pain treatment. This underlines the comprehensive need of research as well as excellent implementation concepts for pain assessment and pain treatment in elderly individuals with dementia.

## 3. Parkinson's Disease and Other Extrapyramidal Disorders

Patients with Parkinson's disease (PD) often experience pain. By now, pain is commonly accepted to represent one of the PD nonmotor symptoms having a remarkable impact on the PD patients' quality of life. While pain was considered initially as an epiphenomenon of the motor impairment characteristic of the disease, the attention toward this symptom has increased in the last ten years. Two main elements led clinical and research efforts to understand the pain mechanisms in PD: (1) the higher prevalence of pain in PD patients compared to that in the healthy elderly subjects and (2) the involvement of nondystonic body parts, which means that pain is possibly linked to the intrinsic pathophysiological mechanisms of the disease.

### 3.1. Pain Frequency and Clinical Features

Pain in PD is commonly assessed according to Ford's scheme [47]. Five different types of pain can be recognized: (1) musculoskeletal, (2) radicular-neuropathic, (3) dystonic, (4) central neuropathic, and (5) akathisia pain. Whether the classical distinction between the nociceptive and the neuropathic pain is used or not, most Parkinsonian patients refer a nociceptive pain, which can be mainly musculoskeletal and visceral. Musculoskeletal pain derives from abnormal postures of the dystonic body parts, rigidity, and akinesia. Visceral pain is often the consequence of the abnormal function of the vegetative nervous system typical of the disease [48]. In PD, an example of neuropathic pain is represented caused by nerve root irritation following the abnormal posture and motor activity of the Parkinsonian patients. However, most papers on PD pain consider also a far less defined “central neuropathic” pain. This is poorly localized and often described as “boring,” “constant,” and “burning” [49]. The classification of this “central pain” is questionable according to the commonly accepted definition of neuropathic pain as being due to a lesion or a disease of the somatosensory system [3]. Although a clear sensory deficit cannot be demonstrated in PD patients with central pain, there are evidences linking this kind of pain to basal ganglia dysfunctions [50] and abnormal nociception [51].

Although several studies investigated the epidemiologic characteristic of pain in PD, the exact prevalence of this disabling symptom is not definitely known, ranging from 30% to 80% [49]. A recent meta-analysis [52] identified 8 studies which met the criteria of the Quality Assessment of Diagnostic Accuracy Studies (QUADAS) tool. In these studies, the average prevalence of pain was 67.6%, ranging from 40% to 85%. As for the location, the pain was prevalently referred to the lower limbs (47.2%) while it involved less frequently the back (14.3%), the upper limbs (13.4%), and the neck/shoulder region (12.4%). The most frequently reported type of pain was the musculoskeletal one (46%) followed by dystonic (19.6%) and radicular (9.1%) pain. Central neuropathic pain was found in 5.6% of PD patients. Interestingly, the only available community study performed in Norway by Beiske et al. [1] reports the highest prevalence of pain in PD as compared to healthy subjects (83% versus 30%). Valkovic et al. [16] have very recently investigated the modifications in the prevalence of pain during the disease progression. Interestingly, all the 4 Parkinson-related types of pain considered in this study (musculoskeletal, dystonic, radicular, and neuropathic) were more prevalent in the advanced stages of PD confirming their strict relationship with the disease.

It should be mentioned that a couple of studies failed in showing a higher prevalence of pain in PD patients compared to that in healthy subjects [53, 54]. In these studies, however, the prevalence of pain was very low in PD patients as compared to other studies or high in healthy subjects thus suggesting an ascertainment bias [15] (Table 2).

### 3.2. Clinical and Instrumental Assessment

Though pain is very prevalent in PD, in most studies rating scales not specific for this disease were used. Only a very recent multicenter study published the first PD pain specific scale [55]. King's Parkinson's Disease Pain Scale (KPPS) is an interview-based scale used to explore the frequency, intensity, and location of each type of PD pain. Moreover, it also rates the pain modifications associated with motor fluctuations in PD.

### 3.3. Possible Pathophysiological Mechanisms

Although the epidemiological data support the linkage between at least certain types of pain and the biological background of the disease, the pathophysiological mechanisms of pain in PD patients are far from being ascertained. According to the current idea, abnormal nociceptive mechanisms, which could predispose patients to develop spontaneous pain, could be found in PD. This is suggested by psychophysical and neurophysiologic studies in PD patients without pain [15]. Reduced thresholds to different pain modalities were found in PD patients without pain compared with the control subjects [17, 51, 56–63]. Moreover, the laser evoked potential (LEP) amplitude assessing the pain matrix function was reduced in the pain-free PD patients [51] even in the early stages of the disease [58]. As for the role of dopamine in the development of PD pain, the available results are not univocal. Indeed, elements suggesting dopamine importance are represented by the more frequent involvement of the affected side in hemi-Parkinson and the pain modifications according to the PD motor fluctuations [14, 64]. However, the psychophysical and neurophysiologic abnormalities shown in PD patients cannot generally recover after dopamine administration [65]. Monoaminergic systems different from the dopaminergic one could be involved in the pathophysiology of pain in PD.

Finally, it is worth mentioning that a reduction of small unmyelinated fibers was reported in PD both in the skin biopsy [66] and in cornea [67]. However, the meaning of the peripheral fiber reduction in the neurophysiological findings is not clear since the last ones seem most dependent on central nervous system modifications.

### 3.4. Current Evidence on Pain Therapeutic Management

No systematic study on pain treatment in PD is currently available. However, it is conceivable that the treatment strongly depends on the type of pain. Broen et al. [52] reviewed 3 studies, which provided some data. According to them, 37.6% of the PD patients use nonopioid analgesic while 13.5% of them use opioids and 11.8% of them use antidepressant and/or anticonvulsive drugs. Kass-Iliyya et al. [67] described an analgesic effect of the deep brain stimulation (DBS) indicated for the improvement of the motor symptoms. Eight patients, having undergone DBS electrode implant within the subthalamic nucleus, showed an increase of the pain threshold when the DBS was switched on as compared to what happened with the stimulator off. Moreover, the authors used the positron emission tomography to investigate the cerebral activity related to central neuropathic pain in PD patients. It was found that the DBS could reduce the brain activity related to pain.

### 3.5. Main Limitations of Present Studies and Future Direction

In conclusion, pain in PD has become an important element to be considered in the clinical practice since it can worsen the general impairment of the patients. In spite of the number of papers published in the last years, there are still some points which should be improved. Firstly, there is still the tendency to consider the different types of PD pain together in many studies. This can prevent the correct identification of the pathophysiological mechanisms and the best treatments that are unlikely to be the same for musculoskeletal, dystonic, and neuropathic pain. Secondly, the KPPS, which represents the first specific PD pain scale, was published in 2016 [55] while pain was previously assessed on the basis of the examiner's experience or by using some questionnaires not specific for PD. This has surely hampered a systematic classification of pain in PD, which represents the mandatory background for any effective treatment.

## 4. Other Extrapyramidal Disorders

Although pain is often reported by patients with extrapyramidal disorders different from PD, there are only few studies dealing with pain in these conditions. Few studies dealt with pain frequency and features in patients with Huntington's disease (HD). Some clinical reports suggested that pain may not be sufficiently manifested and treated in HD patients and that it may be an underestimated problem [68, 69]. In a study conducted by laser evoked potentials, HD patients in the early stages of the disease showed increased LEPs latencies, inversely correlated with their functional capacities [70]. In that study, only 3 out of 28 patients complained of pain despite the presence of possible postural and muscle skeletal abnormalities. This preliminary observation may support the need of more extensive multicentric observational controlled studies. There is a consistent amount of studies indicating that HD patients show a deficit in recognizing negative emotions and pain of others [71, 72]. The impaired processing of negative experiences may thus be supported by an altered influence of basal ganglia on cortical areas devoted to the elaboration of stimuli requesting an aversive motor response as in the case of painful stimuli [73]. The disturbed processing of negative stimuli including pain may interfere with sensory-motor integration [70, 74] and contribute to the global worsening of the disease as suggested by the correlation found between LEPs abnormalities and disability in HD patients [70].

Some data have been collected for Cervical Dystonia (CD), which is characterized by involuntary twisting neck movements and abnormal head postures [75]. Early studies reported pain in around 70% of CD patients [76, 77]. More recently, Charles et al. [78] published epidemiological data from 1,037 CD patients included in a USA registry (Cervical Dystonia Patient Registry for Observation of OnabotulinumtoxinA efficacy—CD PROBE). At baseline, that is, before OnabotulinumtoxinA treatment, 88.9% (922/1037) of the patients reported pain related to CD. In particular, 70.7% (733/1037) reported moderate or severe pain intensity while 29.3% (304/1037) reported no pain or had only a mild pain intensity. Interestingly, the patients with no/mild pain were older than those with moderate/severe pain, while no difference between groups was found in the onset age or the duration of the disease. Unfortunately, no systematic pain classification, including also a possible separation between different types of pain (according to the model reviewed above for pain in PD), has been performed in CD. This contributes to the uncertainty about the pathophysiological mechanisms subtending pain in this condition. Indeed, it is conceivable that pain in CD can be due to the abnormal contraction of the dystonic muscles. However, this possibility has been challenged by the observation that botulinum toxin treatment does not always reduce pain [79, 80]. Moreover, pain is not always correlated with the severity of dystonia in the neck muscles [81]. These elements could lead to hypothesizing a susceptibility of the nociceptive system in CD similar to that demonstrated in PD. Tinazzi et al. [82, 83] recorded LEPs in 20 CD patients by stimulating the skin overlying both painful and nonpainful muscles. They failed to show any abnormality of the nociceptive input processing in these patients making the hypothesis of a central sensitization of the pain matrix in CD unlikely. However, it has to be mentioned that partially different results were obtained in patients with nonpainful hand dystonia by using the contact heat evoked potentials (CHEPs) [84]. Indeed, 6 out of 10 patients showed reduced amplitude of their pain related brain responses to stimulation of the dystonic hand.

Pain was described as a common symptom also in multiple system atrophy (MSA) [85, 86]. This finding was confirmed by a more recent study comparing 21 MSA patients with 65 PD patients [87]. Pain prevalence was similar between MSA (81%) and PD (89%) patients, as well as the scarce response of pain to dopamine administration in both conditions. In MSA, the pathophysiology of pain could be similar to that in PD, involving basal ganglia degeneration. This is suggested by the results of a neurophysiological study exploring pain processing in MSA and PD patients by using the nociceptive withdrawal reflex (NWR) recording [88]. MSA patients showed facilitation of a series of pain responses as compared to healthy subjects. However, no difference was found between MSA and PD patients in terms of neurophysiological abnormalities.

The progressive supranuclear palsy (PSP) is even less investigated than the previously reviewed conditions. The few available data suggest that pain in PSP is far less prevalent than that in PD [87, 88]. However, the only neurophysiological study in PSP showed a lower pain threshold in PSP patients than that in control subjects suggesting that the apparently low prevalence of pain in this condition could be related to the early cognitive impairment of the patients [88].

## 5. Motor Neuron Disease: Amyotrophic Lateral Sclerosis

Amyotrophic lateral sclerosis (ALS), also known as motor neuron disease (MND), is the most common neurodegenerative disorder of the motor system in adults. ALS is characterized by a degeneration of primary motor neurons in the cortex, brainstem, and spinal cord. The amyotrophy (atrophy of muscle fibers) leads to muscular paralysis due to loss of innervating motor neurons. The lateral sclerosis typical of the disease refers to the upper motor neuron axonal loss and the hardening of corticospinal tracts and the resultant gliosis [89, 90]. These changes can lead to a number of debilitating conditions that reflect aberrant functioning in both upper and lower motor neurons. These characteristic features of ALS are also accompanied by a number of secondary conditions that can be just as burdensome as those symptoms directly associated with the disorder. Although degeneration of motor neurons is pivotal in ALS, it is actually considered a multisystemic disorder involving sensory system. A spinocerebellar pathway (Clarke's column) taking origin in the spinal cord segments is consistently affected in pathological studies of ALS, which may underlie the early symptom of impaired balance often reported by patients at diagnosis [90]. In addition, clinicians have long noted minor sensory and autonomic involvement in patients with ALS [91] and small fiber neuropathy was found in skin biopsies in 79% of ALS patients [92]. The link between ALS and frontotemporal dementia represents an extension of ALS as a motor system disease to the frontal and temporal lobes, which are brain areas involved in the expression of thought, planning, personality, and speech, all aspects of brain function that may interfere with pain perception [93]. The complexity of ALS pathophysiology and clinical appearance justifies the need to manage associate symptoms including pain.

### 5.1. Pain Frequency and Clinical Features

Thirty years ago, pain was reported to occur in ASL patients [94]. In the following years, some studies dealt with the frequency of pain in ALS patients, though most of them were conducted in small cohorts of patients in different stages of the disease; in addition, the case control design was sometimes omitted. In most studies, the usual classification in nociceptive and/or neuropathic pain was not used. Ganzini et al. [18] evaluated pain in ALS patients with direct interviews and questionnaires to caregivers. They found that both methods showed high representation of painful symptoms as causes of invalidity (Table 3). A case control study on neuromuscular diseases found that pain was present in 73% of the total population (193 patients) with ALS patients showing the greatest pain interference [19]. The pain was especially located in the back and shoulder, followed by neck, buttock and hip(s), feet, arm(s), and hand(s) [19]. Shoulder pain was also observed in 43 out of 193 ALS patients, independently of age, gender, and phenotype [20].

In a case control study, Chiò et al. [21] found that ALS patients reported pain more frequently than control subjects. Pain was correlated with the disease stage and invalidity. In an observational study on a small ALS series (42 patients), 19 experienced pain, which worsened the quality of life [22]. Rivera et al. [23] used the neuropathic pain scale [95] to study pain in 63 ALS patients in different stages of the disease. They found that about half of patients reported neuropathic pain, which was invalidating since it was present even in the early stages of the disease (Table 1). Pizzimenti et al. [24] observed pain in 72% of 36 ALS patients causing depression and a significant decline of the quality of life. More recently, Wallace et al. [25] specified that in 81% of 41 ALS patients complaining of pain this did not have neuropathic characteristics. In another recent study, 78% of the examined patients complained about pain, which interfered with the quality of life, sleep, and mood [26]. The lack of correlation between severity of pain and disease duration was also reported in this latter study (Table 3). Another study applied the DN4 test for neuropathic pain scoring [96] in 92 ALS patients, who reported only rare symptoms of neuropathic origin [27]. Summarizing, both observational and case control studies reported a high frequency of pain in ALS patients from around 20% in retrospective studies to 80% in case control ones. Characteristics of pain symptoms have been collected with different scales though the most recent studies seemed to suggest the nonneuropathic origin of pain.

### 5.2. Clinical and Instrumental Assessment

As reported above, different scales were used to assess the pain features in ALS patients (Table 3). The short form of BPI was generally applied [97] while only few studies assessed the neuropathic origin of pain with specific questionnaires as DN4 or neuropathic pain scales [96] (Table 3). In most studies, the factors aggravating pain, such as depression and sleep disturbances, were also assessed.

Neurophysiological studies and in particular electromyography and electroneurography are routinely applied for diagnostic purposes to explore motor system in ALS patients [98] while the employment of methods for the specific assessment of nociceptive pathways functions was rarely reported though it would be useful to improve the knowledge about pain pathophysiology and its management. Contact heat evoked potentials were used to explore noxious stimuli conduction along the C-fibers in 60 ALS patients versus 60 controls, and no significant abnormality was found in patients corroborating the hypothesis of an intact nociceptive system [99]. These results were not confirmed in a CO2 laser evoked potentials (LEPs) study conducted in 23 ALS patients [100], who showed an increase in LEPs latencies but also in the amplitude of the earlier latency N1 potential. This is a contradictory result possibly explained by a probable dysfunction of the nociceptive pathways at subcortical level [101], coexisting with enhanced excitability of the nociceptive cortex as a result of motor cortex degeneration. In that study, 19 patients complained about pain, which was usually of the musculoskeletal type supporting the hypothesis that, also in the presence of signs of nociceptive system dysfunction, pain is an indirect consequence of motor impairment [100]. Small fiber involvement was demonstrated by skin biopsy. Weis et al. [92] found a significant reduction in the epidermal nerve fiber density in the distal calf of patients with ALS, which was recently confirmed in another study based on skin biopsy and Quantitative Sensory Testing (QST). In this last study, only patients with spinal onset, but not those with bulbar form, showed an impairment of the thermal sensitivity and distal C afferents [102]. Moreover, a sensitive axonal involvement may be a feature of ALS subtypes.

### 5.3. Possible Pathophysiological Mechanisms

Clinical studies seem to indicate the nonneuropathic origin of pain in the majority of ALS patients though the complexity of this multisystemic degenerative disorder may account for a dysfunction of the nociceptive system at both peripheral and central level. The neuropathic components of ALS-related pain can be present even in the early phases of the disease and worsen the musculoskeletal pain. The presence of the nociceptive afferents dysfunction in ALS is suggested by both skin biopsies and QST especially in the spinal onset phenotype [18, 102]. In addition, the complex interaction between the motor and sensory cortex may cause disinhibition and hyperactivation of sensory functions in order to improve sensory-motor integration in a situation of motor failure [103, 104]. This could explain the increased amplitude of LEPs [100] and SEPs [105] observed in the early stages of the disease.

### 5.4. Current Evidence on Pain Therapeutic Management

A recent Cochrane review [106] reported that there is no evidence from randomized controlled trials about the management of pain in ALS so no guidelines on this important aspect of the disease are available for clinicians. In an observational study, 17 out of 36 ALS patients complaining about pain received treatment with nonsteroidal anti-inflammatory drugs, opioid, or antiepileptics with unspecified results [24]. In another observational study, 63% of 91 ALS patients suffering from pain were under treatment in most of the cases with nonsteroidal anti-inflammatory drugs and nonopioid analgesics [21]. Cramps are currently treated by carbamazepine or phenytoin [107]. In a hospice study, where more than 80% of the patients received the analgesic therapy at least once a day, opioids offered benefit to about 70% of the patients with advanced motoneuronal disease [108, 109]. The treatment of spasticity by intrathecal baclofen may also alleviate pain though this aspect was rarely considered [110]. In addition, although riluzole is actually indicated as the only available disease-modifying medication and confers a little survival advantage, the effects of symptomatic treatment on pain remain unclear [111]. Again, pain associated with ALS is believed to be largely due to immobility. Physiotherapy, stretching, and range of motion exercises used in combination with pharmacotherapies to prevent contractures and reduce cramping and spasticity can be effective for associated pain [107, 112].

### 5.5. Main Limitations of Present Studies and Future Directions

ALS is a complex disorder where pain is currently considered an important but not primary end point in current management. Studies on pain frequency in ALS present the limits to be conducted in single centers in small samples, to rarely have a reliable control population, to be frequently retrospective, and to use usually nonvalidated methods for pain evaluation so the real impact of pain symptoms on the global burden of the disease is still unknown. Neurophysiological examination is limited to standard examination of sensory neurography, and the few studies with neurophysiological techniques exploring the nociceptive and nonnociceptive somatosensory system were conducted in small cohorts of patients in different stages of the disease. Controlled randomized trials on different pain killers are still lacking, and pain was considered only in some studies focusing on the global management of ALS.

Considering the impact of pain on the total outcome of ALS, a systematic clinical approach with specific scale for pain features and invalidity should be used in patients who report painful symptoms. A neurophysiological assessment of sensory functions by means of somatosensory nociceptively and nonnociceptively evoked responses might complete the standard examination [98]. The effects of treatments on pain should be considered, and specific, controlled trials are needed.

Although* Cannabis* may potentially represent a therapeutic opportunity for many ALS symptoms including pain [113, 114], evidence on its efficacy is presently scarce and based only on one controlled study in a small patient group, with negative results on pain release [115]. Angiotensin-converting enzyme inhibitors may be also a potential approach to neurodegenerative disorders and neuropathic pain [116]. In addition, physical therapy and the other nonpharmacological approach would be finalized toward pain symptoms improvement.

## 6. Other Rare Neurodegenerative Conditions

### 6.1. Spinocerebellar Ataxia (SCA)

Among the neurodegenerative hereditary cerebellar ataxia conditions, there are at least 36 different forms of autosomal dominant cerebellar ataxia (ADCA), 20 autosomal recessive cerebellar ataxia conditions, two X-linked ataxia conditions, and several forms of ataxia associated with mitochondrial defects [117].

A number of disease entities present with the ADCA phenotype such as spinocerebellar ataxia (SCA) conditions, dentatorubral-pallidoluysian atrophy, episodic ataxia, and autosomal dominant spastic ataxia. SCA conditions can be divided by the mode of inheritance into autosomal dominant, autosomal recessive, or sporadic conditions. There are many types of spinocerebellar ataxia and about 30 different gene mutations, 22 different genes, and 10 different gene loci have been identified, but the numbers continue to grow [118–120].

The definition of SCA conditions, despite significant progress in their understanding, is still imprecise, but the development of genetic profiling has made genetic classifications possible, which allows estimating the underlying etiology in 60% of the patients [121]. SCA disorders are a group of neurodegenerative disorders with clinical, genetic, and neuropathological heterogeneity being characterized by ataxia and other neurological signs such as oculomotor disturbances, cognitive deficits, pyramidal and extrapyramidal dysfunction, and bulbar, spinal, and peripheral nervous system involvement [122]. The prevalence of ADCA conditions is estimated to be around 3 in 100000, but it is highly variable depending upon the geographical area [123, 124].

As compared with other neurodegenerative disorders such as Parkinson's disease, quantitative and validated assessment tools are less developed [125]. The patients generally experience problems with mobility, usual activities, pain/discomfort, depression/anxiety, and self-care. Different population surveys have shown that 19 to 64% of patients report pain as a problem in selected SCA conditions [126]. In the same study, multivariate analysis revealed three independent predictors of subjective health status: ataxia severity, extent of noncerebellar involvement, and the presence of depressive syndrome. Although pain is not a primary invalidating factor in such patients, it may influence the quality of life as part of depression-related symptoms cohort and noncerebellar features.

In a recent systematic review reporting data from 1062 publications and 12141 patients with different neuromuscular disorders, pain was found to be reported in 1 among 30 SCA sufferers [127]. However, pain may often be underestimated though it can be severe when related to dystonia. In SCA conditions, pain can be misdiagnosed and mistreated but successfully ameliorated by, for example, botulinum toxin therapy [128].

There are currently no cures for SCA and treatments (pharmacological therapy and physiotherapy) target the symptoms such as pain, spasticity, tremor, stiffness, postural balance, gait disabilities, sleep problems, and depression. However, there are some very preliminary and nonvalidated data suggesting the use of umbilical cord mesenchymal stem cells in SCA [129].

### 6.2. Spinal Muscular Atrophy (SMA)

Spinal muscular atrophy (SMA) is an autosomal, recessive, severe neuromuscular, degenerative disease characterized by loss of alpha motor neuron function in the spinal cord resulting in progressive proximal symmetrical muscle weakness often greater in the legs than in arms, atrophy, and paralysis and eventually in impairment of respiration and dysphagia. SMA is the second most common lethal, autosomal, recessive disorder in Caucasians with an incidence of approximately 1/6000 and a carrier frequency of 1/50 [130]. The muscle weakness can cause contracture formation, spinal deformity, limited mobility, and activities of daily living and eventually cause pain.

Engel et al. [131] found chronic pain in most patients classified as “other MND” including the CMT disease, all forms of spinal muscular atrophy, and many forms of mitochondrial and congenital myopathies. Pain was most frequently reported in the legs with a mild intensity (1.3, range 0–6 on the 0–11 numerical scale) [131].

## 7. Conclusions

Neurodegenerative diseases represent a social, medical, and economic problem and constitute a main field of interest for neurologists. Pain may be one of the most debilitating symptoms and a mode to express subjective discomfort and sufferance. Motor and sensory deficit may directly cause pain as in amyotrophic lateral sclerosis, Parkinson's disease, spinal cerebellar ataxia, and hereditary neuropathies where the subjective expression may be limited by the motor impairment. In demented patients, pain may be caused by different factors as age-related muscle skeletal degeneration, immobility, or neurodegeneration in brain areas involved in pain inhibition though subjective sufferance manifestation may be limited by cognitive impairment. The present review outlined a general medical carelessness with regard to pain as attention is especially pointed to the main illness symptoms. However, in many conditions such as Parkinson's disease, amyotrophic lateral sclerosis, and chronic familiar polyneuropathy, pain is largely represented among patients pending its specific assessment. Even in Alzheimer's disease, if special care is provided and specific scales are used, pain appears not to be a secondary problem but instead it appears worthy of full consideration. In rare conditions as Huntington's disease, pain expression may be also limited, and its causes would be underestimated and neglected. As a consequence of the scarce attention generally dedicated to pain, no controlled trials and specific treatment guidelines are available, and pain therapy is generally based on the symptomatic approach by analgesics and anti-inflammatory drugs without a systematic consideration of the causal mechanisms. Current evidences about the relevance of pain in neurodegenerative disorders indicate the opportunity of a full involvement of neurologists in pain management taking into consideration its causes and mechanisms giving special attention to predisposing factors symptoms, employing and validating specific scales performing the clinical and instrumental assessment of sensory functions promoting therapeutic trials by means of pharmacological and nonpharmacological approach. Considering the centrality of pain in individual suffering, the question “Do you feel, or did he/she feel pain?” followed by a careful observation and consideration of the contribution of painful symptoms to the global burden of the disease should be included in the routine assessment of neurodegenerative diseases to finalize the best therapeutic choice.

## Figures and Tables

**Table 1:** Summary of the studies on the prevalence of pain among patients with dementia living at home or in nursing homes. Features and location of pain were not available.

	Study design	Number of patients and controls	Assessment method	Frequency of pain
Barry et al. [7]	Observational	Patients: 75 Controls: 0	Interview with patients and caregivers	*Daily pain* Patients' report: 57% Caregivers' report: 71%

Barry et al. [8]	Observational	Patients: 42 Controls: 0	Interview with patients, nurses, and relatives	*Daily pain* Patients' report: 38% Nurses' report: 69% Relatives' report: 75%

Hunt et al. [9]	Observational	Patients: 802 Controls: 802	Interview with patients	*Bothersome pain* Patients' report: 64% Controls' report: 55%

Werner et al. [10]	Observational	Patients: 141 Controls: 55	Category rating scale	*Pain is experienced* Patients' report: 21% Patients' and Caregivers' report: 46% Controls' report: 48.1

Mäntyselkä et al. [11]	Observational	Patients: 75 Controls: 446	Interview with the patients	*Any pain* Patients' report: 43% Controls' report: 69%

Shega et al. [12]	Observational	Patients: 150 Controls: 0	Interview with patients and caregivers	*Pain right now* Patients' report: 32% Caregivers' report: 52%

Zwakhalen et al. [13]	Observational	Patients: 117 Controls: 0	Observational pain scale	*Experience of pain to some extent* Nurses' observations: 47%

**Table 2:** Summary of studies on the prevalence and features of pain among patients with Parkinson's disease.

	Study design	Number of patients and controls	Assessment methods	Frequency of pain	Feature and location of pain
Nègre-Pagès et al. [14]	Observational	450	Visual analogue scale	278 patients (61.8%)	*PD related (167 patients)* *Other types (111 patients)*

Defazio et al. [15]	Case control	402	Visual analogue scale	*281 patients (69.9%) and 199 controls (62.8%)*	*Nondystonic (267 patients)* *Dystonic and nondystonic (14 patients)*

Beiske et al. [1]	Observational	176	Structured interview (SF-36)	*147 patients (83%)*	Musculoskeletal (103 patients) Dystonic (59 patients) Radicular (0 patients) Central (15 patients)

Valkovic et al. [16]	Observational	100	Brief Pain Inventory Leeds assessment of neuropathic symptoms and signs	*76 patients (76%)*	Musculoskeletal (41%) Radicular (27%) Central (22%) Dystonic (17%) Others (31%)

Tinazzi et al. [17]	Observational	117	Visual analogue scale	*47 patients (40%)*	Dystonic (19 patients) Musculoskeletal (22 patients) Radicular (4 patients) Central (2 patients)

**Table 3:** Summary of studies on the prevalence and features of pain among patients with amyotrophic lateral sclerosis.

	Study design	Number of patients and controls	Assessment methods	Frequency of pain	Feature and location of pain
Ganzini et al. [18]	Observational	100 patients	Interview to patients and caregivers	19% reporting moderate to severe pain	Not reported

Jensen et al. [19]	Observational	193 patients with neuromuscular disease (30 ALS)	Neuropathic pain scale, Brief Pain Inventory, quality of life (SF-36)	60% of ALS patients	“Deep,” “tiring,” “sharp,” and “dull” Localized in the back, leg, shoulder, and neck (total of patients with neuromuscular disease)

Ho et al. [20]	Retrospective	193 patients	Standard medical records	23%	Shoulder pain

Chiò et al. [21]	Case control	160 patients	Brief Pain Inventory	56.9%	Pain more frequent in the extremities

Pagnini et al. [22]	Observational	40 patients	Italian Pain Questionnaire, McGill Quality of Life Questionnaire	51.2%	“Nagging,” “sore,” “periodic,” “annoying,” “exhausting,” “enduring,” “debilitating,” and “worrying”

Rivera et al. [23]	Observational	63	Neuropathic pain scale	50%	Neuropathic pain

Pizzimenti et al. [24]	Observational	36	Neuropathic Pain Symptom Inventory (2 items)	71%	Localized in scapular-humeral area and lower limb

Wallace et al. [25]	Case control	42	Brief Pain Inventory PainDETECT Questionnaire	85%	Nonneuropathic: cramping, aching, tiring, sharp, and tender

Hanisch et al. [26]	Case control	46	Brief Pain Inventory	78%	Cramps

Moisset et al. [27]	Observational	93	DN4 questionnaire	66%	9% neuropathic pain
